# Environmental enrichment reduces displacements and reorganises circadian patterns: a putative indicator of positive welfare in dairy calves

**DOI:** 10.3389/fvets.2026.1842779

**Published:** 2026-08-05

**Authors:** Matthew Thomas, Francesca Occhiuto, Jorge A. Vázquez-Diosdado, Jasmeet Kaler

**Affiliations:** School of Veterinary Medicine and Science, University of Nottingham, Sutton Bonington Campus, Leicestershire, United Kingdom

**Keywords:** aggressive behaviour, biorhythm, dairy calf behaviour, diurnal (circadian) rhythm, environmental enrichment, positive welfare, social displacement

## Abstract

**Introduction:**

Measuring positive welfare in farm animals at scale remains one of the greatest unsolved challenges in animal welfare. Here we demonstrate for the first time that environmental enrichment alters the diurnal activity patterns and aggressive behaviour of pre-weaned dairy calves, suggesting that both could function as automated indicators of an improved welfare state.

**Methods:**

In this quasi-experimental study, ultra-wideband location collar-mounted sensors and automatic milk feeders were used to monitor the behaviour of 300 dairy calves across 21 cohorts, five of which received stationary brushes as environmental enrichment. The diurnality index, defined as the proportion of total activity occurring during daylight hours, was derived from location data, and aggressive interactions were quantified as feeder displacements using a validated algorithm based on visit timing to the feeder.

**Results:**

Enriched calves showed higher diurnality (*β* = 0.26 95% credible interval [0.01, 0.49]) and performed fewer feeder displacements (long duration *β* = -0.25 [-0.49, -0.003]; short duration *β* = -0.26 [-0.51, -0.01]), indicating reduced feeder-directed competition and potentially improved access to milk replacer. Crucially, within the same analytical framework, diurnality decreased during sickness and increased with enrichment.

**Discussion:**

This work provides the first evidence that biological rhythms function as a candidate bidirectional indicator of welfare state, contracting under negative experiences and expanding under positive ones. Alongside this, the reduction in feeder displacements demonstrates that social aggression could also be responsive to positive welfare interventions, together establishing that precision livestock technologies can detect not only welfare deterioration but also its improvement, opening a new frontier in automated welfare monitoring for farm animals.

## Introduction

The capacity to measure positive welfare in animals at a scale remains one of the most important unresolved challenges in welfare science. Positive welfare is characterised by the achievement of positive mental states elicited by pursuing desirable outcomes and having fulfilling experiences (1). While indicators of positive welfare such as play behaviour, cognitive bias and qualitative behaviour assessment exist (2–4), these approaches mostly rely on direct observation and cannot be deployed continuously or at commercial scale (but see Vázquez-Diosdado et al. (5) for automated quantification of play behaviour). Biological rhythms offer a promising alternative. Circadian organisation is increasingly recognised as an indicator of physiological and psychological state across vertebrates, with circadian disruption serving as a marker of allostatic load and chronic stress in mammals (6, 7). In dairy calves, precision livestock technologies have enabled the automated measurement of diurnality defined as the proportion of total locomotor activity occurring during daylight hours (8). Our research has shown that it decreases consistently during disease and acute stress events such as disbudding (8), demonstrating that biological rhythms are responsive to negative welfare experiences and can be captured non-invasively at scale. As diurnality decreases with negative welfare events, it is plausible that a positive welfare intervention may correspondingly increase it, suggesting it could serve as a bidirectional indicator of welfare state, but this has never been tested. Alongside biological rhythms, feeder displacement rates as quantified automatically from the milk feeder have also been shown to decrease during sickness (9), demonstrating that competitive social interactions at the feeder are equally valid indicator of welfare state. Together, diurnality and feeder displacement rate represent a complementary pair of automatically derived welfare measures one capturing the temporal organisation of activity, the other the dynamics of social competition both responsive to welfare deterioration, but neither tested in response to interventions promoting positive welfare.

Environmental enrichment provides an ideal framework within which to test this. The expansion of dairy production has led to increasingly prevalent indoor group housing systems, where confinement in an unstimulating environment can deprive animals of stimuli they are adapted to seek, potentially leading to boredom and maladaptive behaviours (10, 11). Stationary brushes are among the most practical and well-evidenced forms of enrichment for housed cattle, providing a substrate for scratching and grooming behaviours cattle are highly motivated to perform (12), but are largely prevented from expressing indoors, and have been shown to reduce signs of boredom in adult dairy cows (13). Although brush use in pre-weaned calves is less common than in adult cows, calves engage with brushes when provided and show measurable changes including reductions in non-nutritive oral behaviours, indicating an improvement in welfare through the removal of a negative experience, and increases in play behaviour (14, 15). Our previous work demonstrated that enrichment increases daily distance travelled and reduces time spent at the feeder in calves, indicating that its effects extend beyond direct brush interaction to reshape broader patterns of behaviour (15). In older feedlot steers, access to brushes reduced headbutting, suggesting enrichment modulates aggressive social interactions across cattle populations (16). Whether these broader behavioural effects of enrichment extend to diurnal activity patterns and feeder displacements in pre-weaned calves has not been investigated.

In this quasi-experimental study, we used ultra-wideband collar-mounted location sensors and automatic milk feeders to monitor the behaviour of 300 pre-weaned dairy calves across 21 cohorts, five of which received stationary brushes as environmental enrichment. We tested two specific predictions: first, that enriched calves would show higher diurnality (prediction 1), reflecting a shift in biological rhythms toward greater daytime activity inverse to the decrease observed during negative welfare events such as disease; and second, that enriched calves would perform fewer feeder displacements, reflecting reduced competitive pressure at the feeding station (prediction 2). Both outcomes were examined within the same analytical framework that previously captured their deterioration during sickness, enabling a direct test of their response to improved welfare. Confirmation of these predictions would provide the first evidence that diurnality may function as a bidirectional indicator of welfare state and that feeder displacement rates respond to positive welfare interventions, together establishing that precision livestock technologies can detect not only welfare deterioration, but also its improvement, opening a new frontier in the automated monitoring of positive animal welfare.

## Methods

### Ethical approval

The study was carried out between August 2021 and September 2025 at the Centre for Dairy Science Innovation (CSDI) at the University of Nottingham, UK (latitude 52.83871, longitude −1.24972) and approved by the Ethics Committee of the School of Veterinary Medicine and Science, University of Nottingham (unique reference number 1481150603).

### Animals, housing and management

In total 300 calves from 21 cohorts of 10 to 16 calves each were included in the study, with an average of 14.28 (±1.83) animals per group, monitored for an average of 55 days (±15). All the female Holstein Friesian (HO) calves born between June 2021 and July 2025 were included, unless they were in poor health and were at risk of being euthanised or were part of an incompatible study. As this study used data from a previous experimental trial, no *a priori* sample size calculation was conducted for this study. From birth, calves were housed in pairs in straw bedded pens measuring 3 m x 2 m. All the calves were disbudded at least 5 days prior to the start of the trial. Disbudding was performed by trained farm staff using a heated iron and a local anaesthetic (Adrenacaine) was administered 10 min prior to the procedure in accordance with farm protocols. When enough pairs of calves to form a cohort had reached 21 days of age, they were moved to one of two identical group pens of 6 m x10m. The mean age for the calves moving to the group housing was 34.13 (± 5.48) days. Calves were fed with an automatic milk feeder (Forster-Technik, COMPACT smart) with milk replacer (Milkivit Energizer ECN, Trouw Nutrition GB) until weaning. The automatic feeding machine utilized RFID ear tags to identify calves and dispense milk replacer. Milk replacer allocation began at 05:00 a.m. each day and allowed a release of 2 litres every 2 h. In the pair housing total daily allocation began at up to 6 litres per calf at 2 days old, rising gradually to 10 litres per calf per day at 40 days old. Upon moving to the group housing all calves were fed 10 litres of milk replacer per day, with a maximum of 2 litres every 2 h. A step-down weaning process began after 36 days in the group housing, where milk allowance was decreased uniformly for 20 days until calves were fully weaned. In both housings calves had ad-lib access to troughs containing water, concentrates and chopped straw. The barns that the pens were in had open sides to allow air flow and natural lighting. If the building was still dark when the farm staff started their routine checks at 6 a.m. the lights were switched on and were also on in the evenings until 6 p.m.

### Enrichment

**Figure 1:**
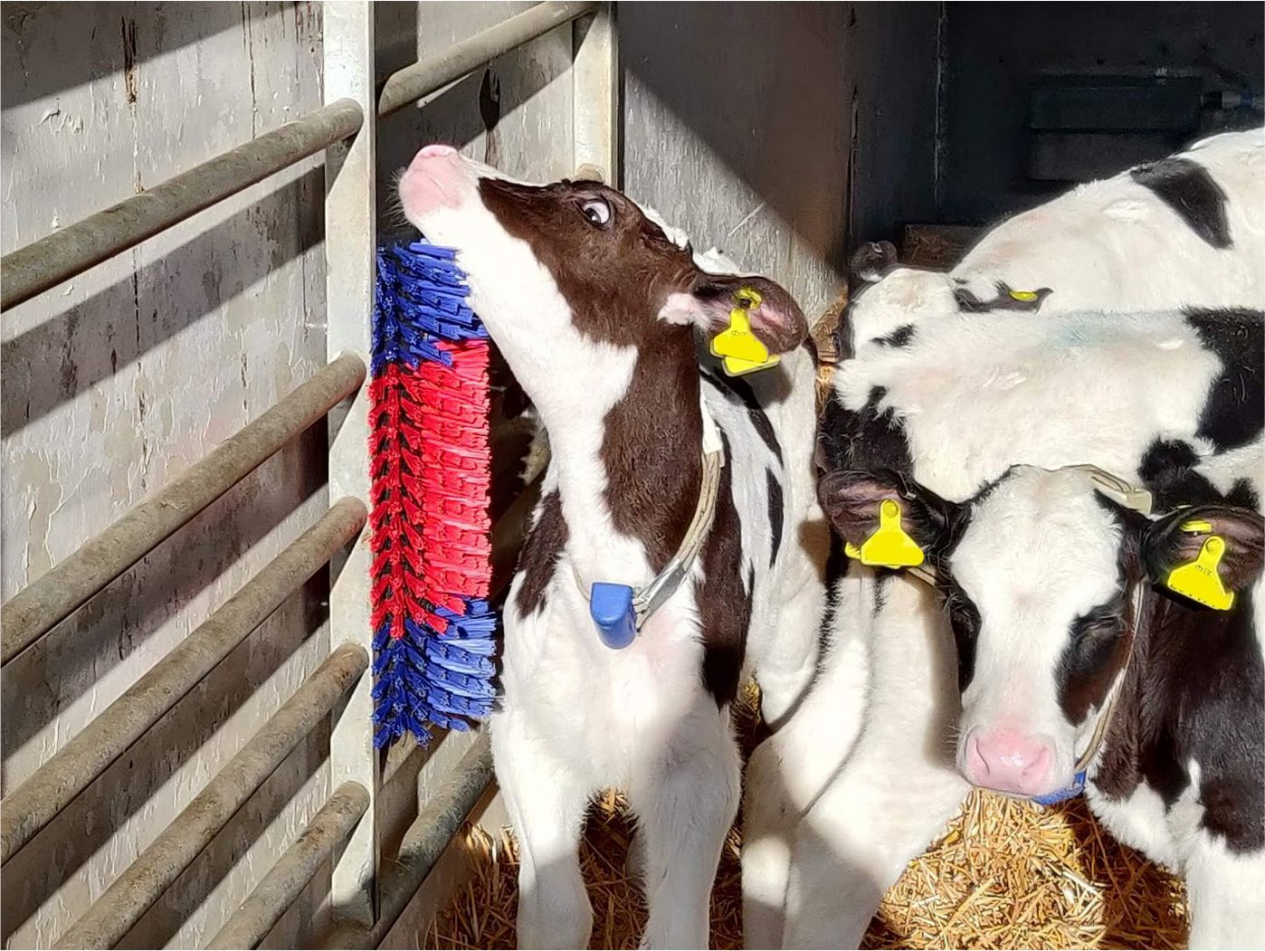
Calf using the brush provided as environmental enrichment in the group pen.

**Figure 2:**
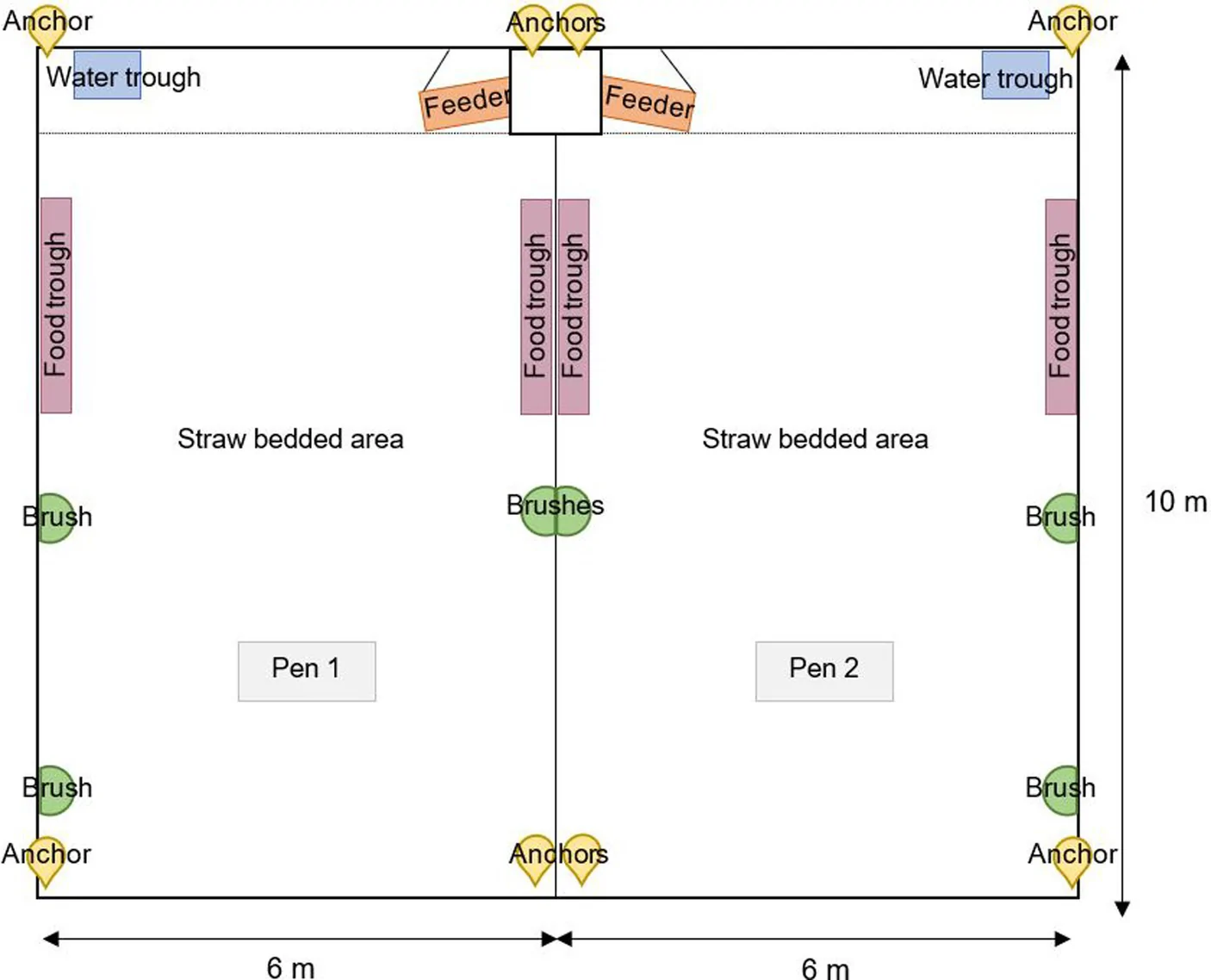
Diagram of the trial pens showing the positions of the key features.

Cohorts were allocated to enrichment or control as part of the study design. Due to operational constraints of the farm 5 cohorts received enrichment and 16 served as controls. Enrichment was provided for the full duration of each cohort’s time in the group housing in the form of three stationary brushes (Kerbl Scratch Brush, Figure 1) fixed to the perimeter of the pen (Figure 2). The brushes were removable, so the control and enriched treatment was balanced between the two pens.

### Sensor data collection and health monitoring

From 4 days old calves were fitted with a collar holding an Ultra-Wideband Sewio Leonardo iMU tag, (Noldus, Wageningen, the Netherlands), which reported the coordinates of each calf with frequency of 1 Hz, in accordance with Occhiuto et al. (17). The location data produced by the tags was validated in the trial pen and the precision was shown to be 0.15 m [0.12–0.28] while the accuracy was 0.17 m [0.13–0.33 m] (17). The coordinates from the sensors were processed into distances by a custom script in R. The Euclidean distance between points was calculated for each second and the data aggregated appropriately by summing over these distances within each day, hereafter called daily distance travelled.

The automatic feeder recorded the visits by each calf and reported the time the individual entered and exited the range of the RFID tag reader. This information was available until the end of the weaning process for all cohorts.

In the group housing pen, calves were scored twice weekly by trained researchers according to a modified version of the Wisconsin-Madison scoring system (18). This health assessment includes scoring the eyes, nose, ear, cough, faecal consistency and rectal temperature with scores from 0 to 3. These individual scores were added to obtain a total Wisconsin score. Daily health scores were obtained by imputing the Wisconsin score, temperature score and cough score between these observations using an exponential moving average. The exponential moving average considered values from 4 days prior to the day to be imputed and 4 days after. This ensured that the algorithm included at least 2 manual scores, while retaining a localised score to accurately reflect the recovery or decline of a calf. Calves with an overall Wisconsin score > 4.5 and/or rectal temperature of >39.4ºC and/or a cough score > 2 were considered to be sick.

Ambient temperature was measured every 2 hours using temperature loggers (ALTA 900 MHz Industrial Humidity Sensors with Probes). These measurements were used to extract an average daily temperature.

### Behavioural parameters

#### Diurnality

The diurnality index (DI) was calculated from the distribution of daily distance travelled by each calf derived from the location data. Diurnality was defined as:


$$\mathrm{DI}=\frac{D-N}{D+N}$$

Where *DI* is the diurnality index, *D* is the total distance travelled within daytime hours and *N* is the total distance travelled within night-time hours for each calendar day. The index ranges between −1 and 1 where the extremes represent nocturnal and diurnal respectively, whereas animals with a DI of 0 would show no clear temporal preference in walking behaviour. Daytime and nighttime were assigned according to average annual sunrise and sunset data retrieved from the National Oceanic and Atmospheric Administration, NOAA (https://gml.noaa.gov/grad/solcalc/sunrise.html) for the latitude and longitude for the CSDI.

#### Displacements

We used our previously developed algorithm for displacement detection based on the feeding patterns and validated using visual observations (9). Within this algorithm, a displacement was considered long duration if the time elapsed between exit and entry was more than 10 and less or equal than 40 s, and short duration if the elapsed time was 10 s or less. Short duration displacements, where the incoming calf entered the feeder almost immediately after the previous calf exited were thought to represent higher urgency interactions where the displacing calf was already waiting in close proximity. Long duration displacements interactions where a calf approached and entered the feeder within a longer window were thought to reflect a less immediate competitive interaction.

### Data analysis

In all models we compared the cohorts with enrichment to those that did not have enrichment. In the diurnality model, observations were taken at a calf-day level, while in the social analysis data was aggregated to cohort-day level in order to measure the effect of enrichment on the total number of displacements for that day across all the calves.

#### Diurnality

We used a Bayesian double hierarchical generalised Gaussian linear model to examine the effects of different covariates, modelling the variation within and between calves separately. The model was fitted using the “brms” package (19) in R 4.5.0 (20). The model included Calf ID and Cohort number as random effects, with age (in months), weaning status (pre-wean, weaning, weaned), month of the year to account for day length and temperature changes (categorical, Jan—Dec), health score (sick, healthy) as fixed effects. In addition, enrichment was added as a binary fixed effect based on whether the calf was in a cohort with exposure to brushes. After excluding any calves or calf-days for missing data, the final dataset contained 298 calves from 21 cohorts with a total of 16,586 calf-days. The response variable, diurnality index, was scaled to give a mean = 0 and sd = 1.

#### Association of enrichment with displacement behaviour

Displacements as measured by the algorithm developed in Vázquez-Diosdado et al. (9) were categorised into two types of displacement, either short or long-duration. For each type of displacement, a separate Bayesian model was used to analyse the effect of enrichment on the pre-weaning data. Models were built using “brms” in R 4.5.0. The outcome for each model was the total number of short or long-duration displacements on that day for the entire cohort. The model included “cohort” as a random effect, with mean temperature, day number, number of animals in the cohort, average age when moved to housing and the number of sick animals as fixed effects. These covariates were all *z*-scaled. Temperature was used in this model instead of month as the environmental covariate most directly relevant to social behaviour (21, 22) and enrichment was also added as a fixed effect in the form of a cohort level binary categorical variable. Each Bayesian model was fit with a negative binomial likelihood to account for the count data, with 4 chains, 5,000 iterations and 2000 burn-in iterations per chain. The model converged with Rhat < 1.05 for all estimates.

## Results

### Effects of enrichment on diurnality

**Figure 3:**
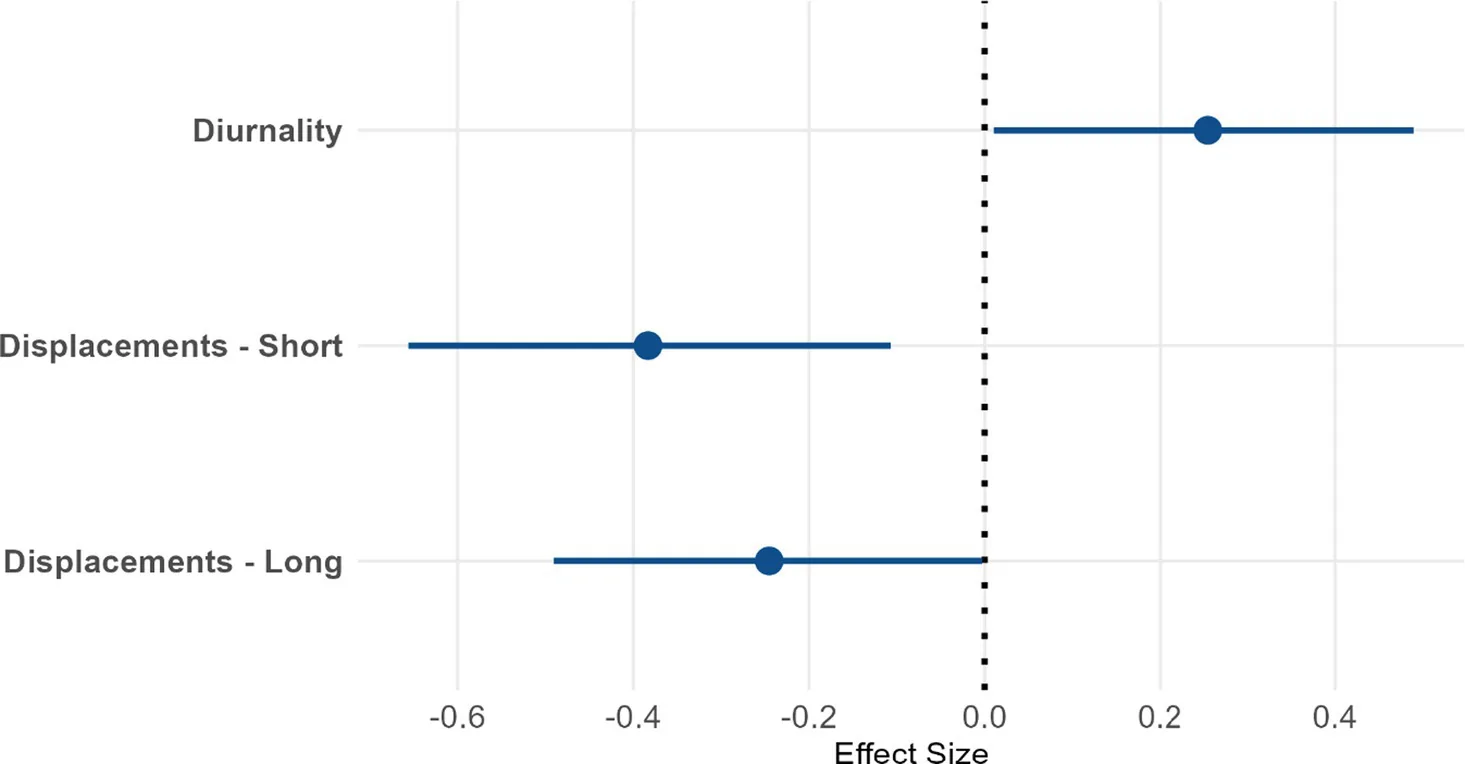
Effect size of enrichment on the diurnality of the calves and on the number of displacements (short and long duration). The lines represent the 95% credible interval.

Calves in this study showed a preference for daytime activity with mean DI = 0.34 with SD = 0.16. Enrichment had a positive effect on diurnality (*β* = 0.26, CI = 0.01, 0.49, Figure 3). The full model output and estimates are reported in Table 1.

**Table 1:** Estimates for random and fixed effects in the diurnality model.

Random effect	SD	95% CI
Calf_ID	0.24	[0.22, 0.27]
Cohort	0.21	[0.15, 0.31]

Fixed effect	Estimate	95% CI
Age (months)	0.19	[0.14, 0.23]
Wean status: weaning	−0.09	[−0.14, −0.04]
Wean status: weaned	−0.30	[−0.38, −0.23]
Month: feb	−0.18	[−0.28, −0.09]
Month: mar	0.16	[0.07, 0.25]
Month: apr	−0.18	[−0.27, −0.07]
Month: may	−0.68	[−0.79, −0.57]
Month: jun	−1.23	[−1.34, −1.11]
Month: jul	−0.44	[−0.56, −0.33]
Month: aug	−0.08	[−0.03, 0.19]
Month: sep	0.38	[0.28, 0.49]
Month: oct	0.31	[0.21, 0.40]
Month: nov	0.17	[0.09, 0.25]
Month: dec	0.08	[0.01, 0.14]
Health: sick	−0.08	[−0.12, −0.03]
Enriched: yes	0.26	[0.01, 0.49]

Calves = 298, cohorts = 21, calf-days = 16,586.

### Effects of the presence of enrichment on long duration displacement behaviour

There were an average of 19.64 (SD = 12.51) long duration displacements per day across all cohorts. There was some heterogeneity across cohorts, demonstrated by the random effect with SD 0.18 (CI = 0.12, 0.27). Enrichment was associated with a reduced number of long-duration displacements (*β* = −0.25, 95% CI = −0.49, −0.003, Figure 3), i.e., approximately 22% reduction in displacement rate, while the number of animals appeared to increase the number of long duration displacements, and they appeared to decrease as the trial progressed. The full model output and estimates are reported in Table 2.

**Table 2:** Estimates for the effects of the presence of the enrichment on the numbers of long duration displacements per day.

Random effect	SD	95% CI
Cohort	0.18	[0.12, 0.27]

Fixed effect	Estimate	95% CI
Enriched: yes	−0.25	[−0.49, −0.003]
Mean temperature	−0.01	[−0.06, 0.05]
Trial day	−0.24	[−0.27, −0.21]
No. of animals	0.31	[0.20, 0.43]
Avg. move age	0.01	[−0.09, 0.11]
No. of sick animals	−0.01	[−0.04, 0.03]

Cohorts: 21, cohort-days: 729.

### Effects of enrichment on short duration displacement behaviour across all cohorts

There were an average of 65.61 (SD = 29.74) short duration displacements per day across all cohorts. Heterogeneity among cohorts was also present here, demonstrated by the random effect with SD 0.26 (CI = 0.18, 0.38). Enriched cohorts exhibited fewer short-duration displacements (*β* = −0.38, CI = −0. 66, −0.11, Figure 3). The number of short duration displacements decreased as the number of sick animals increased and with a higher ambient temperature. The full model output and estimates are reported in Table 3.

**Table 3:** Estimates for the effects of the presence of the enrichment on the numbers of short duration displacements per day.

Random effect	SD	95% CI
Cohort	0.26	[0.18, 0.38]

Fixed effect	Estimate	95% CI
Enriched: yes	−0.26	[−0.51, −0.01]
Mean temperature	−0.06	[−0.10, −0.02]
Trial day	−0.02	[−0.04, 0.002]
No. of animals	0.14	[0.02, 0.26]
Avg. move age	−0.05	[−0.17, 0.07]
No. of sick animals	−0.05	[−0.08, −0.03]

Cohorts: 21, cohort-days: 730.

## Discussion

This study demonstrates for the first time that providing stationary brushes to pre-weaned calves produces a shift in diurnality toward greater daytime activity, and reduces aggressive social interactions at the feeder, both detected non-invasively through precision livestock technologies. Most importantly, the same analytical framework that captured this enrichment-driven increase in diurnality also detected its decrease during sickness, providing the first evidence that diurnality may function as a bidirectional indicator of welfare state responsive to both its deterioration and its improvement. This positions automated monitoring of biological rhythms not merely as a disease detection tool, but as a potential marker of improved welfare. These findings fundamentally extend our understanding of what enrichment does to the lived experience of a calf, demonstrating that a simple, low-cost intervention reshapes not only how calves organise their day, but how they interact socially and thus has implications for how we assess and monitor the welfare of farm animals at scale.

Cattle are mostly diurnal animals and previous studies have confirmed that calves are also mostly active in the daylight hours (8, 23). As per our first prediction, the calves that experienced the brushes were more diurnal than the ones that did not, meaning that the calves had a higher proportion of their total activity during the day. This finding is consistent with our previous work that demonstrated increase in the daily distance travelled in the presence of brushes (15) suggesting brush interaction drives an increase in daytime activity. Although this study did not measure brush use, our previous work in the same system indicates that all calves spend time near the brushes when they are present (15), and therefore are likely to be interacting with them. One possible explanation of the shift in diurnality is that this additional energy expenditure during the day produces compensatory increase in rest and inactivity at night. However, as actual lying, resting and brush use were not measured in the current study this hypothesis needs to be further tested.

Although assessing affective states in non-human animals is challenging due to our inability to objectively detect their emotions (2), some behaviours such as play have been often proposed as indicators of positive welfare due to a decreased frequency during disease events (5) and positive association with environmental enrichment (15). The present findings extend this framework to diurnality. Within the same model diurnality decreased with sickness (in this case respiratory disease, diarrhoea or pyrexia) and increased when the calves had access to enrichment demonstrating that diurnality responds bidirectionally to welfare state. This result is theoretically important as it suggests that diurnality does not merely reflect activity levels but may track the affective experience of the animal. The capacity to detect both deterioration and improvement in welfare automatically and non-invasively has significant implications for development of practical scalable welfare monitoring system on farms, which could further be validated by comparing to established measures of affective states.

As predicted (prediction 2), enriched cohorts had fewer algorithm-detected displacements at the feeder both short duration (approx. 23% reduction) and long duration (Approx 22% reduction) compared to unenriched cohorts. Without access to enrichment, the feeder and water trough represent primary interactive features for calves in the group pen. In this context the higher displacement in unenriched cohorts may reflect lack of alternative behavioural outlet consistent with concept of boredom driven redirected behaviour in indoor housed cows (11). When brushes are available calves have been shown to spend less time in feeder area (15) suggesting enrichment provides alternative behavioural outlet that reduces the extent to which the feeder becomes the focal point of activity in an otherwise unstimulating environment, thereby reducing competitive interactions at the feeder. As the individuals that are lower in the social hierarchy are more likely to be the recipients of agonistic interactions such as displacements (9), the reduction of these interactions would further contribute to an improvement of welfare in these calves. In addition, the fact that calves are being displaced out of the feeder less frequently means that they might also feed more effectively and future work could investigate any effects on weight gain. As in this study we used the feeder data to detect displacements, we were only able to measure the change of displacements at the feeder. It is possible that other types of aggressive behaviour are also affected in calves and future work should investigate this.

It must be noted that an increase in the number of sick calves was accompanied by a reduction in the number of algorithm-detected displacements, meaning that this behaviour alone lacks specificity when used as an indicator of welfare. Accounting for the prevalence of disease within the group, as we did in this study, is therefore necessary to correctly interpret a change in displacements. Rather than a change in affective states, displacements may better represent an outlet for unspent energy, which can decrease both when the energy reserves are depleted by physiological challenges or otherwise occupied by other activities such as brush use.

Overall, these results enhance our understanding of the effects of environmental enrichment on a variety of behaviours. The use of precision livestock technologies allows the non-invasive monitoring of location and feeding patterns to reveal complex behaviours such as diurnality and aggressive social interactions. Our findings demonstrate that these behaviours, which are rarely studied in calves, are affected by environmental enrichment and could therefore be measured automatically to detect changes in welfare. Future studies should build upon this knowledge and measure any impacts of these changes in welfare on production measures and health outcomes. Due to the difficulty in measuring affective states in farm animals, this study highlights the potential of using precision technologies to effectively monitor large groups of animals and detect behavioural changes consistent with improved welfare, while acknowledging that the relationship between these automated measures and the subjective experience of animals requires further validation.

## Data Availability

The raw data supporting the conclusions of this article will be made available by the authors, without undue reservation.
